# Bicuspid Aortic Valve Stenosis and the Effect of Vitamin K2 on Calcification Using ^18^F-Sodium Fluoride Positron Emission Tomography/Magnetic Resonance: The BASIK2 Rationale and Trial Design

**DOI:** 10.3390/nu10040386

**Published:** 2018-03-21

**Authors:** Frederique E. C. M. Peeters, Manouk J. W. van Mourik, Steven J. R. Meex, Jan Bucerius, Simon M. Schalla, Suzanne C. Gerretsen, Casper Mihl, Marc R. Dweck, Leon J. Schurgers, Joachim E. Wildberger, Harry J. G. M. Crijns, Bas L. J. H. Kietselaer

**Affiliations:** 1 Department of Cardiology, Maastricht University Medical Center+ and CARIM, P. Debyelaan 25, 6229 HX Maastricht, The Netherlands; manouk.van.mourik@mumc.nl; (M.J.W.M); hjgm.crijns@mumc.nl (H.J.G.M.C.); b.kietselaer@zuyderland.nl (B.L.J.H.K.); 2 Department of Clinical Chemistry, Maastricht University Medical Center+, P. Debyelaan 25, 6229 HX Maastricht, The Netherlands; steven.meex@mumc.nl; 3 Department of Radiology & Nuclear Medicine, Maastricht University Medical Center+ and CARIM, P. Debyelaan 25, 6229 HX Maastricht, The Netherlands; jan.bucerius@mumc.nl (J.B.); s.gerretsen@mumc.nl (S.C.G.); casper.mihl@mumc.nl (C.M.); j.wildberger@mumc.nl (J.E.W.); 4 Department of Nuclear Medicine University Hospital RWTH Aachen, Pauwelsstraße 30, 52074 Aachen, Germany; 5 Departments of Cardiology and Radiology, Maastricht University Medical Center+ and CARIM, P. Debyelaan 25, 6229 HX Maastricht, The Netherlands; s.schalla@mumc.nl; 6 Centre for Cardiovascular Science, University of Edinburgh, 47 Little France Crescent, Edinburgh EH16 4TJ, UK; marc.dweck@ed.ac.uk; 7 Department of Biochemistry, Maastricht University and CARIM, P.O. Box 616, 6200 MD Maastricht, The Netherlands; l.schurgers@maastrichtuniversity.nl; 8 Department of Cardiology, Zuyderland Medisch Centrum Heerlen/Sittard, Henri Dunantstraat 5, 6419 PC Heerlen, The Netherlands

**Keywords:** bicuspid aortic valve, calcific aortic valve stenosis, vitamin K2, menaquinone-7, PET/MR, ^18^F-NaF

## Abstract

BASIK2 is a prospective, double-blind, randomized placebo-controlled trial investigating the effect of vitamin K2 (menaquinone-7;MK7) on imaging measurements of calcification in the bicuspid aortic valve (BAV) and calcific aortic valve stenosis (CAVS). BAV is associated with early development of CAVS. Pathophysiologic mechanisms are incompletely defined, and the only treatment available is valve replacement upon progression to severe symptomatic stenosis. Matrix Gla protein (MGP) inactivity is suggested to be involved in progression. Being a vitamin K dependent protein, supplementation with MK7 is a pharmacological option for activating MGP and intervening in the progression of CAVS. Forty-four subjects with BAV and mild–moderate CAVS will be included in the study, and baseline ^18^F-sodiumfluoride (^18^F-NaF) positron emission tomography (PET)/ magnetic resonance (MR) and computed tomography (CT) assessments will be performed. Thereafter, subjects will be randomized (1:1) to MK7 (360 mcg/day) or placebo. During an 18-month follow-up period, subjects will visit the hospital every 6 months, undergoing a second ^18^F-NaF PET/MR after 6 months and CT after 6 and 18 months. The primary endpoint is the change in PET/MR ^18^F-NaF uptake (6 months minus baseline) compared to this delta change in the placebo arm. The main secondary endpoints are changes in calcium score (CT), progression of the left ventricularremodeling response and CAVS severity (echocardiography). We will also examine the association between early calcification activity (PET) and later changes in calcium score (CT).

## 1. Introduction

A bicuspid aortic valve (BAV), an aortic valve consisting of two leaflets instead of three, is a common congenital abnormality, occurring in 13.7 per 1000 people in the general population, with a male predominance (3:1) [1,2]. BAV is associated with significant valvular and vascular morbidity and early development of calcific aortic valve stenosis (CAVS) is common. In general, CAVS is characterized by progressive narrowing of the aortic valve and is a known contributor to cardiovascular morbidity and mortality, set to become a major healthcare burden. Clinical trials have not yet presented us with a pharmacological treatment option to allow intervention in the progression of CAVS (Table 1 and Table A1). Therefore, today, the only treatment option for severe CAVS is valve replacement [3]. In patients with BAV, valve replacement is usually indicated between the fourth and sixth decade, which is earlier than in tricuspid aortic valve (TAV) stenosis, in general [4]. This suggests that, in patients with BAV, CAVS shows a more rapid rate of progression [5]. For both BAV and TAV there is an unmet clinical need to delay disease progression. 

Progressive narrowing of the aortic valve is initially caused by lipid infiltration, inflammation and micro-calcification (the very early stages of calcification) and, upon progression, pro-osteogenic and pro-calcific mechanisms dominate, ultimately leading to severe CAVS [6,7,8,9]. These calcific regulatory pathways include Notch, receptor activator of nuclear factor kappa B(RANK)/receptor activator of nuclear factor kappa B ligand (RANKL)/osteoprotegerin (OPG), Wnt/b-catenin and bone morphogenetic proteins (BMPs) [8]. BMP-2 is a key protein of the valvular interstitial cell (VIC) phenotype switching, and thus is highly involved in the progression of calcification. The binding of BMP-2 to its receptor is inhibited by matrix Gla-protein (MGP). Moreover, MGP can directly interact with hydroxyapatite (micro-calcification), inhibiting the growth of hydroxyapatite crystals in vascular tissue [10] and stabilising calcifying protein particles (CPPs) in the circulation [11].

MGP is a vitamin K-dependent protein which needs to undergo carboxylation to become biologically active [10]. In CAVS, the active carboxylated MGP is decreased, thereby inhibiting the ability to inhibit progression of valvular calcification [12]. The beneficial effects of vitamin K in inhibiting vascular calcification have been studied [13,14], but data on the potential effects on CAVS are lacking. Menaquinone-7 (MK7; vitamin K2) has a long half-life (about 3 days [15]) and is reported to have a significantly higher bioavailability and bioactivity in vivo compared to vitamin K1 [16]. 

BASIK2 is being conducted to investigate the effect of vitamin K2, more specifically MK7, on valvular calcification in CAVS, as evaluated by ^18^F-sodiumfluoride (^18^F-NaF) positron emission tomography (PET)/magnetic resonance (MR). PET is a molecular imaging technique that enables the visualization of calcification activity in the valve. The PET tracer, ^18^F-NaF preferentially binds to areas of developing microcalcification [17], predicting where larger macrocalcific deposits will ultimately develop, and, as a consequence, predicting future aortic stenosis progression [18,19]. Integrated MR imaging enables simultaneous evaluation of left ventricular function and structure [20], as well as the visualization of valve morphology and function [21]. It is not hampered by calcification artifacts as seen in computed tomography (CT). Therefore, PET/MR provides incremental information to the standard methods (echocardiography and CT) used to measure aortic valve stenosis and calcification [22]. 

The principal objective of BASIK2 is to provide evidence to support the hypothesis that MK7 inhibits calcification activity in patients with BAV and CAVS. If successful, this would position this simple, safe and naturally occurring agent as the first effective treatment for aortic stenosis and set the foundations for larger phase 3 clinical outcome studies. In addition, the innovative use of sequential ^18^F-NaF PET may help to confirm this hypothesis 6 months after the initiation of therapy. If the change in this parameter predicts the observed changes in CT aortic valve calcification (AVC) and valve hemodynamic at 18 months, then this novel trial design could be used more widely to rapidly and efficiently test the efficacy of other potential therapies in phase 2 clinical trials. 

## 2. Trial Design

The BASIK2 trial is an investigator-initiated, prospective, double blind, randomized, placebo-controlled trial, studying the effects of vitamin K2 (menaquinone-7, MK7) or placebo on the progression of calcification in CAVS using ^18^F-NaF PET/MR in patients with a bicuspid aortic valve and calcific aortic valve stenosis. The study was approved by the institutional review board (Maastricht Academic Hospital and Maastricht University, the Netherlands: NL54600.068.015/METC152045) and conducted according to the principles of the Declaration of Helsinki. The BASIK2 trial is registered in clinicaltrials.gov as NCT02917525. All subjects gave their written informed consent for inclusion before they participated in the study.

In subjects meeting requirements for trial participation an ^18^F-NaF PET/MR and a non-contrast CT will be performed at baseline after providing informed consent. Furthermore, echocardiography and venipuncture will be performed. Thereafter, subjects will be randomized (1:1) to the intervention or control group, receiving an oral dose of 360 micrograms (mcg) menaquinone-7 or placebo respectively (NattoPharma ASA, Hovik, Norway). The total study duration is 18 months, in which subjects will visit the outpatient clinic every six months. After six months, subjects will again undergo PET-MR, and uptake of ^18^F-NaF will be quantified to assess the (difference in) active calcification of the aortic valve and the potential effect of MK7 supplementation. Furthermore, subjects will undergo a (non-contrast) CT after 6 and 18 months. Transthoracic echocardiography and venipuncture will be performed every visit during the follow-up period. Additional clinical information (such as medical history, cardiovascular risk factors, current medication, family history) will be obtained from the electronic hospital charts and will be evaluated every visit (if relevant). 

**Figure 1:**
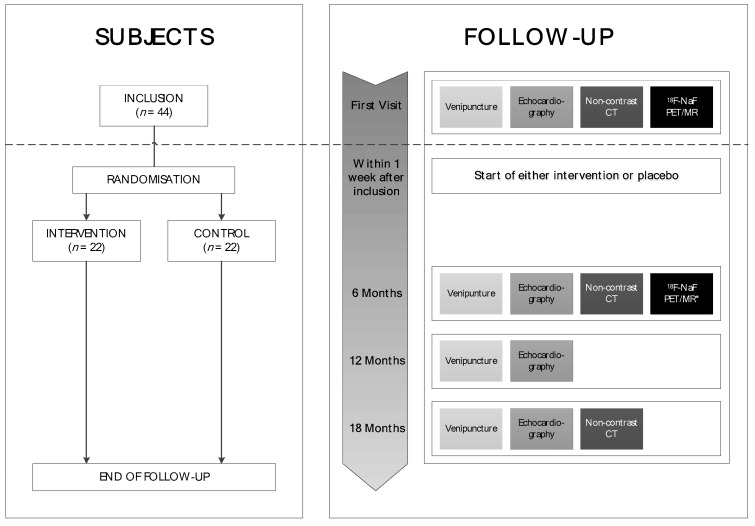
Study flowchart.* Primary endpoint (change from baseline in tracer uptake in the aortic valve by ^18^F-NaF PET/MR at 6 months). Abbrevations: ^18^F-NaF; ^18^F-sodiumfluoride, CT; computed tomography, MR; magnetic resonance, PET; positron emission tomography.

The study flowchart is illustrated in Figure 1. These investigations will enable the evaluation of several effects of MK7 and (the natural course of) progression of CAVS in this population, in addition to the pre-specified primary endpoint. The total study population will consist of 44 patients.

## 3. Inclusion and Exclusion Criteria

A detailed overview of inclusion and exclusion criteria is provided in Table 2. In short, all patients (>18 years) being followed up at the outpatient clinics of the Maastricht University Medical Center+ (MUMC+) with a bicuspid aortic valve (BAV), mild to moderate aortic valve stenosis and calcification confirmed on echocardiography will be screened for eligibility. The presence of BAV will be confirmed using short-axis echocardiographic images and morphology will be determined during systole [26]. Patients who meet any of the exclusion criteria (including standard contra-indications for MR) and those unable to provide written informed consent will not be included. 

## 4. Study Objectives and Statistical Analyses Plan

### 4.1. Primary End Point and Sample Size Calculation

The central aim of the current trial is to assess whether supplementation with menaquinone-7 will slow or even reverse aortic valve calcification activity. Therefore, the primary endpoint is the change in ^18^F-NaF tracer uptake on ^18^F-NaF PET/MR (6 months minus baseline). 

At the time that the current study was designed, literature reporting the specified treatment effect in similar studies was sparse. Therefore, the sample size calculation was based on expected changes in CT calcium scores at the secondary endpoint [22,27,28]. The mean annual calcification progression on CT has been estimated to be 21.7%, with a standard deviation of 19.8% [23]. Considering these premises, the variability of the calcification progression is estimated to be comparable to the standard deviation mentioned above (19.8%). An absolute difference in calcification progression of 20% between the groups is considered a significant effect. With a significance level alpha of 0.05, a power of 80% and an estimated dropout of approximately 25%, 44 patients will be required to detect a difference between the treatment groups (~22 subjects each) [29]. This power calculation is conservative since change in CT calcium score is considered less sensitive than changes in ^18^F-NaF uptake [18], and the calculated number of individuals is expected to afford more power to demonstrate a treatment effect for the primary endpoint (^18^F-NaF uptake). ^18^F-NaF tracer uptake was shown to be present in patients with CAVS in regions overlying, adjacent to and remote from existing valvular calcification [30], and a recent study provided the first preliminary evidence that ^18^F-NaF is a very sensitive marker of progression of aortic valve disease [18]. The six month time window between baseline and follow up measurement with molecular PET imaging is also rather conservative and was derived from earlier studies investigating the effect of short-term statin therapy on vascular inflammation and calcification on fluorodeoxyglucose (FDG) PET/CT, showing a significant reduction in tracer uptake after 3 months of treatment [31,32].

Since it is known that progression of aortic valve stenosis is not a linear process, but rather shows a trend towards an increasing progression rate in advanced disease [33], patients with less than mild aortic valve stenosis and patients with severe calcified aortic valve stenosis at baseline will be excluded. Patients with a bicuspid aortic valve have an increased risk for aortic valve replacement from approximately the fourth decade in life [4], suggesting a more rapid rate of progression, possibly due to altered hemodynamic circumstances [5]. 

### 4.2. Secondary Endpoints

Secondary objectives include the following: change from baseline in calcium score of the aortic valve measured by CT after 6 and 18 months, the correlation between tracer uptake after 6 months and calcium score by CT after 18 months, change from baseline in echocardiographic parameters depicting the progression of CAVS, the response of left ventricular (LV) function to CAVS on echocardiography, and the change from baseline in left ventricular function, left ventricular mass, aortic distensibility and aortic flow on MR after 6 months. Furthermore, the LV response to aortic valve stenosis, as measured by changes in resting cardiac troponin and NTproBNP will be investigated, as well as associations between changes in biomarkers and progression of calcification after 6, 12 and 18 months.

Data from all included patients will be used for the analyses. In the case of exclusion of a patient during the trial, data from the patient will be collected and included in the analyses up until their exclusion.

### 4.3. Additional Analyses

The main outcome parameter (change in calcification activity between the MK7-treated arm and placebo) will be presented as a continuous variable. The mean difference in calcification activity between treatment arms will be expressed as the difference between ^18^F-NaF uptake during follow-up and ^18^F-NaF uptake at baseline. The CT calcium score will be presented as a continuous variable, and the calcification (score) progression will be expressed as the mean difference (calcium score (aortic valve) during follow-up minus calcium score (aortic valve) at baseline) and will be presented as a dichotomous variable (rapid progression and slow progression).

Data will be analyzed based on the intention-to-treat principle. Baseline and follow-up categorical variables will be expressed as percentages and continuous variables as means ± standard deviations. The independent *t*-test or Mann–Whitney U test will be used to test differences between normally-distributed continuous variables and continuous variables not showing a normal distribution, respectively. A paired *t*-test or Wilcoxon signed rank test will be applied when appropriate. Categorical variables will be tested using the Fisher’s exact or Chi square test. A two-sided significance level of 5% will be considered to be statistically significant. 

Univariate analysis and multiple regression analysis will be used to investigate the existence of significant predictor(s) for the outcome variable—calcification progression. 

## 5. Study Procedures

### 5.1. PET and MR Imaging

Combined PET/MR scans will be performed at inclusion and after 6 months of follow-up using a full-integrated Tesla PET-MR scanner (Siemens Biograph Mmr^TM^, Siemens Healthineers, Forchheim, Germany). A dose of 185 MBq of NaF will be injected intravenously. After 30 min, (non-contrast) MR scanning will be started. PET data acquisition will be started 60 min after intravenous administration of the radiopharmacon. Dixon-based MR images will be used for attenuation correction.

Heart and large vessel anatomy will be determined using a T1-weighted black blood sequence (transversal and oblique sagittal plane) turbo spin echo sequence, prospectively triggered (average repetition time (TR)/echo time (TE): 740 ms/27 ms, resolution 1.3 × 1.3 × 8.0 mm). Cine-MR views of the heart in the horizontal and vertical long axes, short axes and left ventricular outflow tract will be acquired according to standard clinical protocols to obtain ventricular volume, mass and function, and 3–5 slices of cross sectional cine images at the level of the aortic root will be acquired to obtain valvular anatomy and function (all cine images: balanced fast field echo sequence, retrospectively triggered. TR/TE/flip angle: 41.28 ms/1.51 ms/50°, resolution 1.3 × 1.3 × 8.0 mm). Flow imaging will be performed at the level of the aortic valve and the ascending aorta. Analyses of source images will be performed using dedicated software (Syngo.via^TM^, Siemens Healthineers, Forchheim, Germany). PET signal quantification will be performed by delineating regions of interest (ROI) using both PET and MR images. Moreover, (non-contrast) CT images will be used to localize regions of macrocalcification.

### 5.2. Computed Tomography (CT) Imaging

A breath-held, non-contrast, enhanced CT scan will be performed at inclusion and during the visits at 6 and 18 months of follow-up to determine calcification of the aortic valve and the thoracic aorta. These scans will be performed using a third generation, dual-source CT-scanner (Somatom Definition Force, Siemens Healthineers, Forchheim, Germany). The scan protocol for calcium scoring will be performed at a tube voltage of 120 kV, reference quality tube current of 80 mAs, 2 × 192 × 0.6 mm collimation, a gantry rotation time of 0.25 s and a pitch value of 3.2. Calcification quantification (mass, volume and score) of the aortic valve and the thoracic aorta will be determined using dedicated post-processing software (Syngo.via, Siemens Healthineers, Forchheim, Germany) [27]. Calcium localized from the sinotubular junction to the end of scan range, or up to the origin of the brachiocephalic artery, is considered to be in the ascending aorta. Calcium present distal from the origin of the left subclavian artery up to the diaphragm is considered to be localized in the descending aorta. Quantification will be performed by two observers, both blinded to medical data. In the case of ambiguity, consensus will be reached by discussion/in the presence of a third observer.

### 5.3. Echocardiography

Transthoracic echocardiographic examinations will be performed every 6 months. All parameters (presented in Table 3) are part of the regular echocardiographic examination and will be assessed according to EAE/ASE guidelines.

### 5.4. Laboratory Assessments

Blood sampling will be conducted by standard venipuncture during all study visits. Standard hematological parameters (hemoglobin, hematocrit, thrombocytes, leucocytes) and differentiation will be evaluated. Additional samples will be stored at −80 **°**C for future biomarker analyses investigating kidney function, vitamin K status and calcification inhibitor concentrations over time. Moreover, the association between biomarkers and calcification/aortic valve stenosis, left ventricular response and (long term) diastolic function will be investigated in future analyses. 

### 5.5. Randomization and Study Intervention

Subjects will be randomized after the initial scans in a 1:1 fashion, by an independent investigator, to the (1) intervention group (MK7) or (2) placebo group (Figure 1). Block randomization (4 or 6 subjects per block) will be assembled to safeguard equal allocation of subjects to the treatment groups. 

### 5.6. Study Intervention

Patients in the intervention group will receive a capsule containing 360 mcg of menaquinone-7 (MK7, NattoPharma ASA, Oslo, Norway) daily for 18 months. Capsules consist of synthetic MK7 (bioequivalent to soy and natural chickpea MenaQ7) [41]. The choice to use MK7 is based on its longer half-life and its favorable extra-hepatic distribution compared to other forms of vitamin K2 [16]. The dose to be used in this trial was established in a dose-finding study, in which a positive dose-dependent effect of menaquinone-7 on MGP- and osteocalcin-carboxylation was found. Non-functional MGP was decreased most effectively using a daily dose of 360 mcg MK7 [42,43,44]. Furthermore, MK7 does not cause a hypercoagulable state and is well-tolerated [45]. 

The placebo capsule does not differ from the MK7 capsule with regard to shape, taste and additives, but does not contain MK7. 

Patients receive a pre-specified number of tablets at each visit. The next visit, patients will hand the leftover tablets to the investigator who will provide the patient with the next pre-specified number of tablets. Compliance will be monitored at each visit by performing and registering a pill count. Furthermore, vitamin K status and concentration of dephosphorylated uncarboxylated MGP (dp-ucMGP) over time will be determined at the end of the study.

## 6. Summary

The BASIK2 study is a proof of concept trial that will provide us with information on calcium activity in the aortic valve and the potential effect of supplementation with vitamin K2 (more specifically; MK7). This trial bears the potential to open novel avenues for future large scale randomized controlled trials to intervene in the progression of CAVS.

## Figures and Tables

**Table 1:** Overview of randomized controlled trials, performed with various pharmacological interventions, to halt progression of calcification in aortic valve stenosis.

Intervention	Trial	Year or Clinicaltrials.gov Number	No. of Patients	Main Inclusion Criteria	Primary Endpoint	Conclusion
Atorvastatin vs. placebo	SALTIRE (Scottish Aortic Stenosis and Lipid Lowering Trial: Impact on Regression) [23]	2005	155	Patients (>18 years) with aortic valve stenosis (V_max_ ≥ 2.5 m/s) and aortic valve calcifications, without indications for AVR	Calcium score and V_max_ progression in atorvastatin, arm vs. placebo (using echocardiography and cardiac CT at baseline, 12 and 24 months)	Atorvastatin had no effect on the rate of change in V_max_ or valvular calcification
Atorvastatin vs. placebo	TASS (Tyrolean Aortic Stenosis Study) [24]	2008	47	Patients (>18 years) with aortic valve stenosis (mean gradient ≥15 mmHg, V_max_ ≥ 2.0 m/s) and aortic valve calcifications, without indications for AVR	Calcium score and mean pressure gradient progression in atorvastatin arm vs. placebo (using echocardiography and cardiac CT at baseline, 12 and 24 months)	Atorvastatin did not reduce progression of CAVS based on mean pressure gradient and aortic valve calcification
Vitamin K1	Slower progress of aortic valve calcification with vitamin K supplementation. Results from a prospective interventional proof-of-concept study [25]	2017	99	Patients with asymptomatic or mildly symptomatic aortic valve calcification (V_max_ > 2.0 m/s), without indications for aortic valve replacement	Difference in progression of aortic valve calcification between the vitamin K arm and the placebo arm (using cardiac CT at 1 year)	Vitamin K might decelerate the progression of aortic valve calcification, measured by cardiac CT when compared to placebo.
PCSK9 inhibitor vs. placebo	PCSK9 inhibitors in the progression of aortic stenosis	NCT03051360	140	Patients (>18 years) with mild to moderate aortic valve stenosis	Calcium score progression in the PCSK9 treated arm vs. placebo arm (using cardiac CT and NaF PET at 2 years)	Not available
Niacin vs. placebo	EAVaLL (Early Aortic Valve Lipoprotein(a) lowering trial)	NCT02109614	238	Patients (51–84 years) with presence of aortic sclerosis or mild aortic stenosis (AVA > 1.5 cm^2^, mean gradient 25 mmHg) and high Lp(a) (>50 mg/dL)	Calcium score progression in the niacin arm compared to the placebo arm (using cardiac CT at 2 years)	Not available
Alendronic acid vs. placebo; Denosumab vs. placebo	SALTIRE II and RANKL inhibition in aortic stenosis (Study investigating the effect of drugs used to treat osteoporosis on the progression of calcific aortic stenosis)	NCT02132026	150	Patients (>50 years) with aortic valve stenosis based on echocardiography (V_max_ > 2.5 m/s and grade 2–4 calcification), without indications for valve replacement surgery	Change in aortic valve calcium score (using CT at baseline, 6 months and 2 years)	Not available
Ataciguat vs. placebo	A Study Evaluating the Effects of Ataciguat (HMR1766) on Aortic Valve Calcification (CAVS)	NCT02481258	35	Patients (>50 years) with mild to moderate aortic valve stenosis/calcification (1.0 < AVA < 2.0 cm^2^, calcium level > 300 AU and LVEF > 50%)	Change in aortic valve calcium between the HMR1766 arm vs. the placebo arm (using CT at 6 and 12 months)	Not available
Phytine	CALCIFICA (Value of oral phytate in the prevention of progression of cardiovascular calcifications)	NCT01000233	250	Patients (>18 years) with calcium in the aortic valve (characterized by Rosenhek score grade 2/3 on echocardiography)	Calcium in the aortic valve and in the coronary arteries in the phytine arm vs. the placebo arm (using CT at 24 months)	Not available

AVA: aortic valve area, AVR: aortic valve replacement, AU: Agatston units, CMR: cardiac magnetic resonance, CT: computed tomography, Lp(a): lipoprotein(a), LVEF: left ventricular ejection fraction, LVM: left ventricular mass, MGP: matrix Gla protein, PCSK9: proprotein convertase subtilisin/kexin type 9, NaF PET: sodium fluoride positron emission tomography, RAS: renin–angiotensin system, V_max_: peak velocity.

**Table 2:** Eligibility criteria.

**Inclusion criteria**
✓ Age > 18 years
✓ Presence of bicuspid aortic valve
✓ Calcified mild to moderate aortic valve stenosis (mean gradient < 40 mmHg, maximum gradient between 25–64 mmHg or V_max_ between 2.5–4 m/s)
**Exclusion criteria**
- Absence of bicuspid aortic valve
- Absence of calcified aortic valve stenosis (mean gradient < 10 mmHg, Vmax < 2.5 m/s or AVA 3–4 cm^2^)
- Presence of severe aortic valve stenosis (mean gradient > 40 mmHg, maximum gradient > 64 mmHg or AVA < 1.0 cm^2^)
- Aortic valve replacement or repair (scheduled)
- Accepted atrial fibrillation
- Use of vitamin K antagonists
- Malignant disease < 2 years (except non-melanoma skin cancer, or in situ carcinoma of the cervix)
- Life expectancy < 2 years
- Present pregnancy or wish for near future pregnancy
- Claustrophobia
- Metallic implant (neurostimulator, cochlear implant, vascular clip)
- Pacemaker or ICD
- Adipositas per magna

V_max_: peak jet velocity; AVA: aortic valve area; ICD: implantable cardiac defibrillator.

**Table 3:** Echocardiographic parameters.

Anatomy and function AoV [34,35,36]
Diameter LVOT, aortic sinus, STJ, ascending aorta
Systolic LV function and dimension [37,38]
Filling pressure and LV diastolic function [39]
RV function [40]

AoV: aortic valve, LVOT: left ventricular outflow tract, STJ: sinotubular junction, LV: left ventricle, RV: right ventricle.

## Appendix A

**Table A1:** Overview of randomized controlled trials performed with various pharmacological interventions with primary endpoints other than calcification in aortic valve stenosis.

Intervention	Trial	Year or Clinicaltrials.gov Number	No. of Patients	Main Inclusion Criteria	Primary Endpoint	Conclusion
Simvastatin + ezetimibe vs. placebo	SEAS (Simvastatin and Ezetimibe Aortic Stenosis) [46]	2008	1873	Patients (45–85 years) with asymptomatic mild to moderate aortic valve stenosis (V_max_ 2.5–4.0 m/s)	Major cardiovascular events	No difference in occurrence of major cardiovascular events
Rosuvastatin vs. placebo	ASTRONOMER (Aortic Stenosis Progression Observation: Measuring Effects of Rosuvastatin) [47]	2010	269	Patients (18–82 years) with asymptomatic mild to moderate aortic valve stenosis (V_max_ 2.5–4.0 m/s)	Peak gradient and AVA progression in rosuvastatin arm vs. placebo (using echocardiography at baseline and annual measurements)	Rosuvastatin had no effect on the progression of aortic valve stenosis based on peak gradient and AVA
Rosuvastatin vs. placebo	PROCAS (Progression of Stenosis in Adult Patients With Congenital Aortic Stenosis) [48]	2011	63	Patients (18–45 years) with asymptomatic congenital aortic valve stenosis (V_max_ ≥ 2.5 m/s)	Aortic valve stenosis progression based on V_max_ in rosuvastatin arm vs. placebo (using echocardiography at baseline and annual measurements)	Rosuvastatin had no effect on the progression of congenital aortic valve stenosis (based on V_max_, mean gradient and AVA)
Fluvastatin vs. placebo	AORTICA 1 (Randomized Study to Evaluate the Efficacy of Fluvastatin on Inflammatory Markers in Patients With Aortic Stenosis)	NCT00404287	164	Patients (>18 years) with asymptomatic aortic valve stenosis (V_max_ > 2 m/s)	Changes in CRP (mg/dL) concentrations at 12 months	Not available
Fluvastatin vs. placebo	Statin Therapy in Asymptomatic Aortic Stenosis	NCT00176410	100	Patients (21–80 years) with asymptomatic mild to moderate aortic valve stenosis (V_max_ > 2.5 m/s, 0.8 <AVA <1.5 cm^2^)	Progression of aortic valve stenosis and hemodynamic parameters (using TTE and catheterization at 24 months)	Not available
Ramipril vs. placebo	RIAS (a prospective, double-blind, randomized controlled trial of the angiotensin-converting enzyme inhibitor ramipril in aortic stenosis) [49]	2015	100	Patients (>18 years) with asymptomatic moderate to severe aortic valve stenosis (valve area < 1.5 cm^2^ or V_max_ > 3.0 m/s) without indications for valve replacement surgery	Change in LVM in the ramipril arm vs. the placebo arm (using CMR at baseline at 6 months and 1 year)	Modest (but significant) difference in LVM between the two groups after 1 year (regression of LVM in the ramipril arm vs. increased LVM in the placebo arm)
Captopril and trandolapril vs. placebo	ACCESS (Acute Haemodynamic Effects of Treatment With Angiotensin Converting Enzyme (ACE)-inhibitors in Patients With Symptomatic Aortic Stenosis	NCT00252317	64	Patients (>18 years) with asymptomatic and symptomatic severe aortic valve stenosis (AVA < 1.0 cm^2^)	Improvement of haemodynamic parameters after 8 weeks of treatment with ACE-inhibitor vs. placebo	Not available
Eplerenone vs. placebo	ZEST (A randomized trial of the aldosterone-receptor antagonist, eplerenone, in asymptomatic moderate–severe aortic stenosis) [50]	2008	65	Patients with asymptomatic moderate to severe aortic valve stenosis (V_max_ > 3.0 m/s) with ejection fraction > 50%, without indications for valve replacement surgery	Delay of onset of LV systolic dysfunction or reduction of progression of LV hypertrophy in the eplerenone arm vs. placebo (using CMR)	Eplerenone did not show a clear effect on primary endpoints.
Candesartan vs. placebo	Is blockade of the renin–angiotensin system able to reverse the structural and functional remodeling of the left ventricle in severe aortic stenosis? [51]	2015	51	Patients (>18 years) with severe aortic valve stenosis referred for valve replacement surgery	Changes in LV structure and function and improvement of exercise capacity in the eplerenone arm vs. placebo (at 5 months)	Candesartan did not have favorable effects on the left ventricle or exercise tolerance.
Candesartan vs. placebo	ROCK-AS (The Potential of Candesartan to Retard the Progression of Aortic Stenosis)	NCT00699452	120	Patients (>18 years) with clinically symptomatic severe aortic valve stenosis, not treated with ACE-inhibitors or AT1R antagonists	Inflammation in the valves at 3–5 months	Not available
Fimasartan vs. placebo	ALFA (A Randomized Trial of Angiotensin Receptor bLocker, Fimasarta, in Aortic Stenosis)	NCT01589380	100	Patients (20–75 years), with moderate to severe (asymptomatic) aortic valve stenosis (V_max_ 3.0–4.5 m/s, mean gradient 25–49 mmHg or AVA 0.76–1.5 cm^2^), able to undergo cardiopulmonary exercise testing	Change in VO_2max_ during cardiopulmonary exercise testing at 1 year	Not available
Tadalafil vs. placebo	ASPEN (Aortic stenosis and phosphodiesterase type 5 inhibition: a pilot study)	NCT01275339	56	Patients (>18 years) with moderate to severe aortic valve stenosis (AVA < 1.5 cm^2^), without indications for valve replacement surgery	Change in LVM (using CMR at 6 months), change in diastolic function (using tissue Doppler e’ at 12 weeks and 6 months) and change in LV longitudinal peak systolic strain (using echocardiography at 12 weeks and 6 months)	Not available.

ACE: angiotensin-converting-enzyme, AT1R: Type 1 angiotensin II receptor, AVA: aortic valve area, CMR: cardiac magnetic resonance, CRP: C-reactive protein, CT: computed tomography, LVEF: left ventricular ejection fraction, LVM: left ventricular mass, RAS: renin-angiotensin system, TTE: transthoracic echocardiography, V_max_: peak velocity.
